# PD-L1 Expression in Mesenchymal Stem/Stromal Cells: Impacts on Innate and Adaptive Immunity, Therapeutic Potential, and Biomarker Utility

**DOI:** 10.3390/ijms27104362

**Published:** 2026-05-14

**Authors:** Luna Rahr Futtrup, Anaïs Marie Julie Møller, Amalie Sjøgren, Bjarne Kuno Møller

**Affiliations:** 1 Center for Gene and Cellular Therapy, Department of Clinical Immunology, Aarhus University Hospital, Palle Juul-Jensens Boulevard 99, DK-8200 Aarhus N, Denmark; luna.rahr.futtrup@gmail.com (L.R.F.); amaliesjoegren3@gmail.com (A.S.); bjamoell@rm.dk (B.K.M.); 2 Comparative Medicine Lab, Department of Clinical Medicine, Aarhus University, Palle Juul-Jensens Boulevard 5, DK-8200 Aarhus N, Denmark; 3 Department of Clinical Medicine, Aarhus University, Palle Juul-Jensens Boulevard 99, DK-8200 Aarhus N, Denmark

**Keywords:** mesenchymal stem/stromal cells (MSCs), programmed death-ligand 1 (PD-L1), immunomodulation, immune checkpoints, biomarkers, cell therapy

## Abstract

Mesenchymal stem/stromal cells (MSCs) are multipotent progenitor cells with potent immunomodulatory properties, making them attractive candidates for treating inflammatory and autoimmune diseases. A key mediator of MSC-induced immunosuppression is programmed death-ligand 1 (PD-L1), a checkpoint molecule that interacts with PD-1 on immune cells to regulate immune responses and promote tolerance. This review synthesizes current evidence on the role of PD-L1 expression in MSCs, emphasizing its effects on both the innate and adaptive immune systems, its therapeutic potential, and its utility as a biomarker for MSC potency and clinical efficacy. We examine how PD-L1 modulates T cell activation, dendritic cell maturation, macrophage polarization, and cytokine profiles, including its role in exosomal contexts. Additionally, we highlight its synergistic interactions with other immune checkpoints and discuss its dual function as both a therapeutic effector and a dynamic biomarker. Finally, we explore its relevance in clinical contexts such as autoimmune diseases, graft-versus-host disease, sepsis, and transplantation and conclude with a discussion of challenges and future directions in harnessing PD-L1 for MSC-based therapies.

## 1. Introduction

Mesenchymal stem/stromal cells (MSCs) are non-hematopoietic, multipotent progenitor cells capable of differentiating into various mesodermal lineages. Beyond their regenerative potential, MSCs exhibit strong immunomodulatory properties, which have been extensively studied in the context of autoimmune diseases, graft-versus-host disease (GvHD), and tissue repair [1,2]. These effects are mediated through both soluble factors and direct cell–cell interactions, with programmed death-ligand 1 (PD-L1) emerging as a key checkpoint molecule in MSC-mediated immune regulation [3,4].

PD-L1 (CD274) is a transmembrane protein belonging to the B7 family, that binds to programmed death-1 (PD-1) on T cells, B cells, and myeloid cells, leading to suppression of proliferation, cytokine production, and cytotoxic activity. While PD-L1 is well known for its role in tumor immune evasion, its expression in MSCs has garnered attention for its ability to suppress immune responses and promote tolerance in non-cancerous contexts [5,6].

In contrast to prior literature, this review provides a comprehensive and differentiated perspective on PD-L1 in MSCs. Existing reviews have primarily focused on the canonical role of PD-L1 in T-cell suppression and its cytokine-driven induction, particularly emphasizing IFN-γ-mediated upregulation and broad immunosuppressive effects on adaptive immunity [7]. Similarly, broader immune checkpoint-oriented MSC reviews highlight multi-pathway immunoregulation but do not specifically dissect PD-L1-centric mechanisms, its regulation, or its translational implications [8,9].

Here, we extend beyond these frameworks by integrating several underexplored dimensions of PD-L1 biology. First, we provide an integrated analysis of PD-L1 across both cellular and exosomal compartments, underscoring the emerging functional relevance of PD-L1–positive small extracellular vesicles (sEVs). Second, we position PD-L1 as both a functional mediator and a predictive biomarker of MSC potency and clinical efficacy, with particular attention to implications for manufacturing and quality control. Third, we offer a mechanistic and context-dependent evaluation of PD-L1 in sepsis, highlighting its dynamic and potentially divergent roles across hyperinflammatory and immunosuppressive disease phases. Collectively, this work establishes a more nuanced and translationally oriented framework for understanding and leveraging PD-L1 in MSC-based therapies.

## 2. PD-L1 Expression and Regulation in MSCs

### 2.1. Basal and Inducible Expression

PD-L1 expression in MSCs is both constitutive and inducible, depending on the tissue source and environmental stimuli. Umbilical cord-derived MSCs (UC-MSCs) and adipose-derived MSCs (AD-MSCs) often exhibit higher basal PD-L1 levels compared to bone marrow-derived MSCs (BM-MSCs) [3]. This variability is attributed to differences in the immunological niche and the intrinsic transcriptional landscape of MSCs from distinct sources. De la Rosa-Ruiz et al. (2019) demonstrated that MSCs derived from dental tissues also possess unique immunoregulatory properties, including differential effects on T cell suppression, suggesting that PD-L1 expression and function may vary across less commonly studied MSC sources [10].

Inflammatory cytokines such as interferon-gamma (IFN-γ), tumor necrosis factor-alpha (TNF-α), and interleukin-1β (IL-1β) significantly upregulate PD-L1 expression in MSCs, enhancing their immunosuppressive capacity. This inducibility is critical for MSCs to respond dynamically to immune activation and inflammation [11].

The interaction between PD-L1-expressing cells and T cells through the PD-L1/PD-1 axis is well established. Beyond direct cytokine-mediated regulation, MSCs can enhance PD-L1 expression in surrounding cells through paracrine signaling. Aboulkheyr et al. (2021) showed that MSC-derived CCL5 upregulates PD-L1 in breast cancer cells, extending MSC influence on immune checkpoints beyond their intrinsic phenotype [12].

MSCs additionally release PD-L1–enriched small extracellular vesicles (sEVs). Wang et al. (2025) reported that these vesicles can fuse with T cells, directly inhibiting their proliferation [13]. In addition, proteolytic cleavage of membrane-bound PD-L1 generates soluble PD-L1 (sPD-L1), which itself exerts immunosuppressive activity. Davies et al. (2017) demonstrated that pro-inflammatory cytokines drive the release of both sPD-L1 and sPD-L2, which are sufficient to suppress T-cell activation and induce durable hyporesponsiveness [14].

Together, these findings highlight the diverse mechanisms by which MSCs exploit PD-L1/PD-1 signaling—through direct expression, paracrine chemokine secretion, vesicle-mediated transfer, and soluble forms—broadening their immunomodulatory repertoire [15].

### 2.2. Signaling Pathways and Transcriptional Control

**Figure 1:**
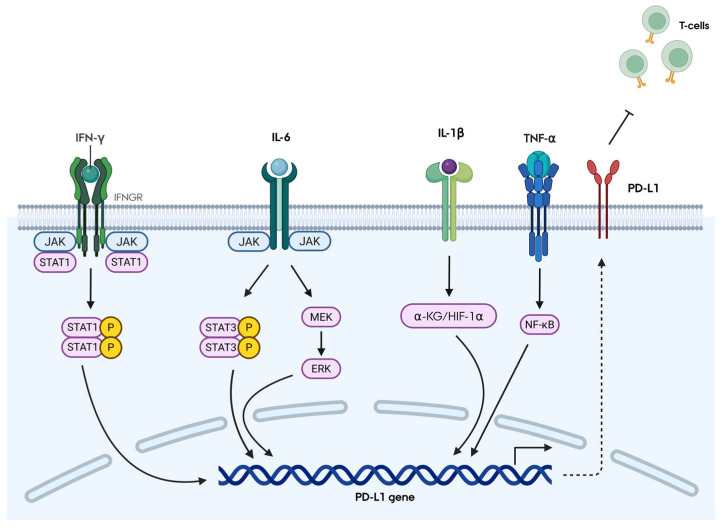
Cytokine-mediated regulation of PD-L1 expression in mesenchymal stromal cells (MSCs). Inflammatory cytokines induce PD-L1 transcription in MSCs via distinct signaling pathways. IFN-γ activates JAK/STAT1, IL-6 signals through JAK/STAT3 and MEK/ERK, IL-1β engages the α-KG/HIF-1α axis, and TNF-α activates NF-κB. These pathways converge to upregulate PD-L1 expression, with IFN-γ acting as the most potent inducer.

The transcriptional regulation of PD-L1 in MSCs is primarily governed by inflammatory and oncogenic stimuli, most notably IFN-γ, TNF-α, IL-1β, and IL-6 (Figure 1). Each cytokine engages distinct intracellular signaling pathways to induce PD-L1 expression. IFN-γ, secreted by activated T cells, binds to IFNGR and activates the Janus Kinase (JAK)/Signal Transducer and Activator of Transcription 1 (STAT1) axis, resulting in robust PD-L1 induction. IL-6 can drive PD-L1 expression via multiple mechanisms, including JAK/STAT3, STAT3/c-Myc/miR-25-3p, and MEK/ERK signaling. IL-1β induces PD-L1 through α-ketoglutarate/hypoxia-inducible factor-1α axis. In contrast, TNF-α predominantly promotes PD-L1 transcription through Nuclear Factor Kappa-light-chain-enhancer of activated B cells (NF-κB) activation [7]. Evidence from several studies, including Jang et al. (2018), Grinnemo et al. (2019), and Behm et al. (2020), highlight IFN-γ as the most potent inducer of PD-L1 in MSCs [4,15,16].

Beyond cytokine-mediated signaling, recent studies have uncovered novel transcriptional regulators of PD-L1 in MSCs. Osawa et al. (2024) identified Paired Related Homeobox 1 (PRRX1) as a key regulator, with overexpression of PRRX1 isoforms significantly enhancing PD-L1 expression and thereby suggesting a role in strengthening MSC immunomodulatory function [17]. Furthermore, transcription factors such as Interferon Regulatory Factor 1 (IRF1) and NF-κB have been identified as key transcriptional regulators of PD-L1 expression in UM-MSCs, by binding to the promoter region of the PD-L1 gene enhancing transcription [11]. These findings indicate that PD-L1 expression is not only shaped by inflammatory signals but also finely tuned by environmental stressors and intrinsic transcriptional networks. Together, these findings highlight a complex but convergent regulatory network, with IFN-γ–STAT1 signaling as the dominant axis, modulated by additional transcriptional inputs and environmental cues.

### 2.3. Post-Translational Modifications

Recent studies have revealed that PD-L1 expression and function in MSCs are regulated not only at the transcriptional level but also through post-translational modifications. Strauch et al. (2020) demonstrated that inflammatory licensing leads to enhanced PD-L1 cell surface expression and secretion, accompanied by increased N-glycosylation [18]. Expanding on these findings, accumulating evidence from both MSCs and other biological systems underscores N-glycosylation as a critical determinant of PD-L1 stability, intracellular trafficking, and functional interaction with PD-1 [18]. In MSCs, enhanced N-glycosylation facilitates efficient transport of PD-L1 to the cell surface and promotes its secretion, suggesting a key role in regulating its bioavailability. Complementary insights from cancer biology further show that glycosylation protects PD-L1 from proteasomal degradation and preserves its PD-1 binding capacity [9]. Together, these findings extend the current understanding of PD-L1 regulation in MSCs by highlighting N-glycosylation as a functionally relevant layer of control with direct implications for MSC immunomodulatory potency.

### 2.4. MicroRNA Regulation

MicroRNAs also play a role in regulating PD-L1 expression in MSCs although this area remains relatively underexplored. Chen et al. (2018) [19] showed that miR-17-5p negatively regulates PD-L1 expression in BM-MSCs. Downregulation of miR-17-5p in MSCs cultured with serum from liver allograft models led to increased PD-L1 expression, suggesting a mechanism by which MSCs adapt to inflammatory environments to enhance immune tolerance [19].

Notably, miR-17-5p remains the only microRNA directly validated to regulate PD-L1 in MSCs to date. However, evidence from non-MSC systems indicates that additional microRNAs may contribute to PD-L1 regulation. For example, miR-34a and members of the miR-200 family have been shown to modulate PD-L1 stability and surface expression in cancer cells, pointing to conserved regulatory pathways that could extend to MSC biology [9]. These observations highlight a potential and underscore the need for further investigation into microRNA-mediated regulation of PD-L1 in this context.

## 3. Effects on the Innate Immune System

**Figure 2:**
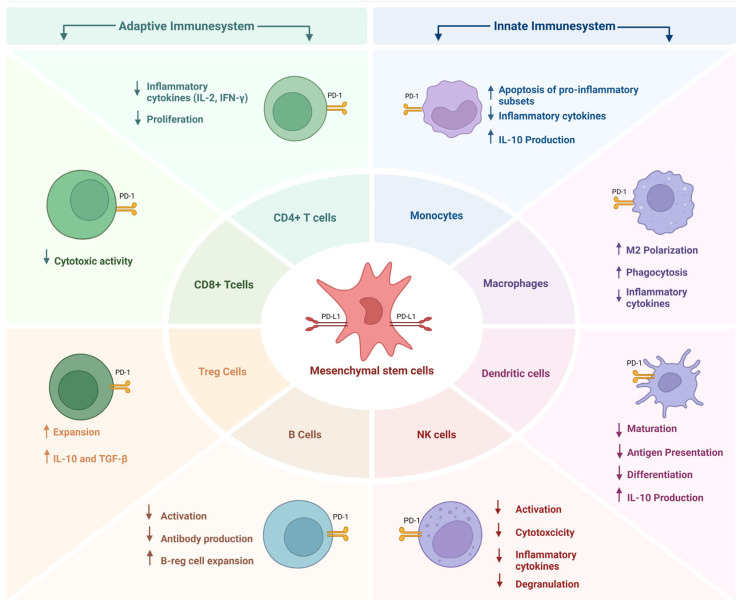
PD-L1-mediated immunomodulatory effects of mesenchymal stem cells. Using the PD-1/PD-L1 axis, MSCs can regulate innate and adaptive immune responses. PD-L1-expressing MSCs suppress CD4^+^ and CD8^+^ T cell activation and cytokine production while promoting regulatory T cell expansion. They inhibit B cell activation and antibody production, impair dendritic cell maturation and antigen presentation. In the innate compartment, PD-L1-expressing MSCs skew monocytes/macrophages toward anti-inflammatory phenotypes and enhance IL-10 production and reduce NK cell cytotoxicity. Collectively they promote an immunosuppressive microenvironment.

MSCs exert profound effects on the innate immune system, primarily through their expression of PD-L1 and secretion of immunomodulatory factors. These interactions influence the behavior of dendritic cells (DCs), macrophages, monocytes, and natural killer (NK) cells, contributing to the resolution of inflammation and the establishment of immune tolerance (Figure 2).

### 3.1. Dendritic Cells

DCs are professional antigen-presenting cells essential for the initiation of adaptive immune responses. MSCs have been shown to suppress both the differentiation and maturation of DCs, resulting in reduced expression of co-stimulatory molecules such as CD80 and CD86, and diminished capacity to activate T cells [8,20]. This immunosuppressive effect is mediated, in part, by the upregulation of PD-L1 on MSCs, which is associated with enhanced suppression of DC function [8,21]. 

MSCs also modulate the cytokine profile of DCs, decreasing the secretion of proinflammatory cytokines and increasing the production of IL-10, thereby promoting a more tolerogenic DC phenotype [8]. In addition to direct cell–cell interactions, MSCs exert their effects through soluble mediators. Exposure to inflammatory cytokines induces MSCs to upregulate PD-L1 and indoleamine 2,3-dioxygenase (IDO), both of which contribute to the suppression of DC activation and function [4,22]. These mechanisms collectively enable MSCs to attenuate the antigen-presenting capacity of DCs and dampen the initiation of adaptive immune responses.

### 3.2. Macrophages and Monocytes

Macrophages are highly plastic cells that can adopt pro-inflammatory (M1) or anti-inflammatory (M2) phenotypes depending on environmental cues. MSCs have been shown to promote M2 polarization, a process that is enhanced by PD-L1 expression. Holopainen et al. (2020) [23] further showed that MSC secretome enhanced the phagocytic activity of macrophages and promoted the expression of M2 markers such as CD206 and IL-10. These changes were associated with increased PD-L1 expression on macrophages, suggesting a feedback loop in which MSCs and macrophages reinforce each other’s immunosuppressive functions [23]. 

Goncalves et al. (2017) [24] demonstrated that MSC-derived membrane particles selectively targeted CD14^+^CD16^+^ monocytes, inducing apoptosis and reducing the frequency of inflammatory monocyte subsets. This effect was mediated by PD-L1 and other immunomodulatory molecules such as prostaglandin E2 (PGE2) and transforming growth factor-beta (TGF-β) [24]. Generally, MSCs have been found to decrease the secretion of pro-inflammatory cytokines and increase the secretion of IL-10 in monocytes. Studies also found that they inhibit the monocyte-induced proliferation of T cells [8]. It is, however, important to note that the effects are mechanistically complex and cannot be attributed to PD-L1 alone. It is therefore essential to distinguish between direct PD-L1-mediated interactions—where MSC-expressed PD-L1 engages PD-1 on macrophages or monocytes—and indirect immunomodulatory effects mediated by soluble factors such as PGE2, TGF-β, and IL-10.

### 3.3. Natural Killer Cells

NK cells are cytotoxic lymphocytes integral to early defense against infections and malignant transformations, mediating their effect through direct cytolysis and the secretion of pro-inflammatory cytokines, such as IFN-γ and granzyme B [25]. MSCs exert potent immunomodulatory effect on NK-cells suppressing their activation, proliferation and effector functions both through direct cell-to-cell contacts and paracrine mechanisms [26,27]. 

Co-culture experiments have demonstrated that BM-MSCs significantly downregulate NK cell activation markers including CD69, NKp30 and NKp44, and inhibit the secretion of INF-γ and granzyme B. This suppression is mediated through soluble factors such as IDO and PGE2 as well as direct cell to cell contact [28]. Importantly, the PD-L1 expression on MSCs has also been shown to inhibit NK cell activation and cytotoxicity through the engagement of PD-1 on NK cells. AD-MSCs from obese individuals, which express higher levels of PD-L1, have been reported to suppress NK-cell function and contribute to immune evasion within adipose tissue [6]. 

Previous studies have shown that activation of the PD-L1/PD-1 axis on NK cells impairs cytokine production and degranulation [29,30]. Although direct studies on MSCs are limited, it is reasonable to infer that the PD-L1 expression on MSCs would likewise promote this axis. Additional studies are needed to clarify the extent to which MSC–NK interactions are mediated through this checkpoint axis. The MSC-mediated suppression of NK cell activity is particularly relevant in transplantation and autoimmune disease context, where excessive NK cell activation can cause tissue damage and drive GVHD [28].

## 4. Effects on the Adaptive Immune System

MSCs exert significant influence over the adaptive immune system, particularly through their expression of PD-L1 and secretion of immunomodulatory molecules. These effects are most pronounced in their interactions with T cells and B cells, which are central to antigen-specific immune responses (Figure 2). 

### 4.1. T Cell Modulation

T cells are central targets of MSC-mediated immunosuppression, with PD-L1 playing a critical role in suppressing their activation, proliferation, and effector functions. Zhou et al. (2018) demonstrated that AD-MSCs suppress CD4^+^ and CD8^+^ T cell subsets via PD-L1/PD-1 interactions, reducing NF-κB activation and cytokine secretion of pro-inflammatory cytokines such as IFN-γ and IL-2 [3]. 

Jang et al. (2018) further confirmed that IFN-γ suppression is mediated through STAT1 signaling, rather than PI3K/Akt pathways, highlighting the importance of inflammatory priming in enhancing MSC immunosuppressive function [15]. 

MSC-mediated suppression of T cell responses is not limited to effector subsets. Guan et al. (2018) demonstrated that PD-L1 and IDO1 expression in IFN-γ-licensed MSCs correlated with their ability to induce regulatory T cells (Tregs), which are essential for maintaining immune homeostasis [1]. These Tregs express FoxP3 and secrete IL-10 and TGF-β, contributing to the resolution of inflammation and prevention of autoimmunity. Moreover, MSCs can modulate T cell survival and apoptosis. He et al. (2022) [29] reported that MSCs in lymph nodes promote T cell survival and activation via the MCP-1/PD-L1 axis. Taken together, these seemingly divergent effects reflect the context-dependent nature of PD-L1 signaling. In early inflammatory responses, PD-L1 primarily acts to restrain excessive T-cell activation and cytokine production, thereby limiting tissue damage. In contrast, within specialized anatomical niches such as lymph nodes, PD-L1 can instead support T-cell survival and controlled activation by modulating the strength and duration of T-cell receptor signaling, as illustrated by the MCP-1/PD-L1 axis [29]. This suggests that PD-L1 does not function as a uniformly suppressive molecule, but rather fine-tunes T-cell responses according to tissue context and the phase of the immune reaction.

### 4.2. B Cell Regulation

Although less extensively studied than T cells, B cells are also modulated by PD-L1-expressing MSCs. In collagen-induced arthritis models, Hu et al. (2021) [31] showed that PD-L1-transfected MSCs reduced B cell activation and antibody production, thereby mitigating autoimmune responses. This suppression was associated with decreased expression of CD19 and reduced levels of pathogenic autoantibodies [31]. 

In addition to suppressing B cell effector functions, MSC interactions with B cells also promote the expansion of regulatory B cells (Bregs), which secrete IL-10 and contribute to immune tolerance. In a mice model studying liver fibrosis, Feng et al., 2024 found that MSCs significantly reduced B-cell activation and infiltration, while also inhibiting the release of pro-inflammatory cytokines [32].

However, it is important to note that the mechanisms underlying MSC-induced B cell modulation remain incompletely defined. While PD-L1 may contribute to these effects, particularly in suppressing B cell activation, the induction of regulatory B cells and broader inhibition of B cell function may involve additional pathways.

### 4.3. T Cell Exhaustion and Checkpoint Crosstalk

In chronic inflammation and cancer, T cells often exhibit an exhausted phenotype characterized by upregulation of multiple inhibitory receptors. PD-L1-expressing MSCs may either contribute to or mitigate this exhaustion depending on the inflammatory context. Zhou et al. (2018) showed that MSCs suppress T cell subsets via both PD-L1/PD-1 and Gal-9/T-cell Immunoglobulin and Mucin-domain containing-3 (TIM-3) pathways, indicating a synergistic effect in immune regulation [3]. In sepsis and chronic infection, elevated PD-L1 expression on MSCs and immune cells has been associated with T cell exhaustion and impaired immune responses [2].

## 5. Therapeutic Applications of PD-L1-Expressing MSCs

The immunomodulatory properties of MSCs, particularly those mediated by PD-L1 expression, have been harnessed in a variety of therapeutic contexts, including autoimmune diseases, transplantation, graft-versus-host disease (GvHD), and infectious or inflammatory conditions. Mechanistically, PD-L1 equips MSCs with a versatile immunoregulatory toolkit, operating across multiple layers of control, including transcriptional and post-transcriptional regulation, vesicle-mediated delivery, and context-dependent interactions with both innate and adaptive immune cells. Through these coordinated pathways, PD-L1 enables MSCs to recalibrate immune activation, suppress excessive inflammation, promote tolerogenic phenotypes, and modulate T- and B-cell responses in a tissue-specific manner. This integrated mechanistic framework provides a strong rationale for the therapeutic use of PD-L1-expressing MSCs. 

By linking molecular regulation to functional immune outcomes, it becomes evident that PD-L1 is not merely a marker of MSC immunopotency but a key effector driving their capacity to restore immune balance. Accordingly, MSC-based therapies exploiting PD-L1 activity hold promise in clinical settings where controlled immunosuppression or induction of tolerance is required.

### 5.1. Autoimmune Diseases

Autoimmune diseases are characterized by dysregulated immune responses against self-antigens, leading to chronic inflammation and tissue damage. MSCs have shown promise in modulating these responses through PD-L1-dependent mechanisms. In a collagen-induced arthritis model, Hu et al. (2021) demonstrated that PD-L1-transfected MSCs ameliorated joint inflammation, reduced pro-inflammatory cytokine expression, and increased the frequency of Tregs and Bregs, resulting in improved histological outcomes and reduced disease severity [31]. 

Similarly, in experimental autoimmune encephalomyelitis (EAE), a model for multiple sclerosis, Kurte et al. (2018) [33] showed that MSCs modulate Th17 cell responses via the IL-17/IL-17RA axis and PD-L1 expression. This interaction led to a decreased infiltration of inflammatory cells into the central nervous system and improved clinical scores [33]. 

In autoimmune hepatitis models, Wharton’s Jelly (WJ)-MSCs with high PD-L1 expression demonstrated superior therapeutic efficacy, including reduced inflammation and enhanced Treg induction [34]. 

These findings support PD-L1 as a selection criterion for MSC-based therapies. MSCs suppress autoreactive lymphocytes and promote regulatory subsets, showing potential in conditions like rheumatoid arthritis and type 1 diabetes [29,35]. Exosomal PD-L1 derived from MSCs has shown promise in modulating immune responses in a rheumatoid arthritis mouse model, offering a cell-free alternative with targeted delivery and reduced systemic toxicity [13].

### 5.2. Graft-Versus-Host Disease (GvHD)

GvHD is a major complication of allogeneic hematopoietic stem cell transplantation, resulting from donor T cells attacking host tissues. MSCs have been used to treat steroid-refractory GvHD due to their immunosuppressive properties. Li et al. (2021) [36] reported that sEVs derived from PD-L1-enriched MSCs suppressed T cell activation, promoted Treg differentiation and improved clinical outcomes in GvHD patients. The rapid increase in circulating PD-L1^+^ sEVs following MSC infusion correlated with reduced inflammatory markers and improved survival [36]. These findings suggest that PD-L1 expression on MSCs and their sEVs may serve as both a therapeutic mechanism and a biomarker for treatment efficacy in GvHD. Moreover, MSCs may reduce the need for prolonged immunosuppression, thereby minimizing the risk of infections and other complications.

### 5.3. Transplantation and Allograft Survival

In solid organ transplantation, immune rejection remains a significant challenge. MSCs have been investigated for their ability to promote graft tolerance and prolong allograft survival. Mardomi et al. (2021) [37] demonstrated that PD-L1 overexpression in MSC-derived cells protected against immune rejection in a murine skin transplant model. This protection was associated with reduced infiltration of CD8^+^ T cells and increased expression of immunoregulatory molecules such as IL-10 and TGF-β [37]. 

Wang et al. (2019) showed that infusion of IFN-γ-conditioned MSCs upregulated PD-L1 and delayed the rejection of vascularized composite allografts [38]. These findings support the use of PD-L1-enhanced MSCs as adjunctive therapy in transplantation to modulate host immune responses and improve graft outcomes. Lynch et al. (2020) further demonstrated that TGF-β1-licensed murine MSCs, which exhibit elevated PD-L1 expression, significantly improved corneal allograft survival and reduced immune rejection, reinforcing the therapeutic relevance of PD-L1 upregulation in transplant settings [39].

### 5.4. Sepsis and Infectious Diseases

Sepsis is a life-threatening syndrome marked by a systemic, dysregulated response to infection. The early phase involves strong innate immune activation via PAMPs and DAMPs, triggering pro-inflammatory cytokine release. Patients who survive this initial hyperinflammatory state either recover or progress into a prolonged immunosuppressive phase, characterized by opportunistic infections and latent viral reactivation [40]. The immunosuppressive phase is defined by T cell apoptosis, reduced cytokine production, and functional exhaustion of effector cells [2].

Sepsis continues to be associated with unacceptably high mortality rates, suggesting that current therapeutic strategies—including antimicrobial therapy, fluid resuscitation, and supportive care—remain insufficient. MSCs have emerged as promising therapies in sepsis, with preclinical studies in multiple animal models demonstrating that MSCs exert anti-inflammatory, immunomodulatory and antimicrobial effects, positioning them as unique candidates for the treatment of sepsis and septic shock [40,41]. 

MSCs rebalance immune responses by polarizing macrophages from a pro-inflammatory M1 to an anti-inflammatory M2 phenotype via PGE2 secretion (Keane et al., 2017 [40]). This shift reduces neutrophil transendothelial migration and cytokine-driven tissue damage while enhancing phagocytic function. Neutrophil depletion abolishes MSC protection, highlighting their central role in immunoregulation [40]. MSCs enhance macrophage and monocyte function through increased phagocytosis and paracrine signaling, contributing to pathogen clearance and immune regulation. MSCs can also induce regulatory T cell (Treg) expansion, a process implicated in the dual role of dampening hyperinflammation while supporting pathogen clearance, ultimately improving survival in sepsis models [40]. 

MSCs enhance antimicrobial defense by promoting bacterial phagocytosis and secreting antimicrobial peptides [40,41]. In animal models, BM- and UC-MSCs reduced E. coli-induced lung injury and bacterial load. MSC-derived sEVs replicate these effects, emphasizing paracrine signaling. In addition to immune reprogramming MSCs contribute to host recovery by promoting tissue repair. They restore epithelial and endothelial barriers compromised during septic injury, thereby limiting pathogen translocation and further inflammation [40].

While most preclinical work has focused on the hyperinflammatory phase of sepsis, fewer studies have addressed the later immunosuppressive phase. When looking at the immunosuppressive phase, the PD-1/PD-L1 axis has emerged as a central driver of this state. Elevated PD-L1 expression on immune cells and MSCs correlates with disease severity and immune suppression in septic patients [2]. Emerging evidence suggests that obesity may exacerbate this mechanism. Eljaafari et al. (2021) demonstrated that AD-MSCs from obese individuals express high PD-L1, impairing T and NK cell responses, potentially worsening sepsis outcomes [6]. It is important to note that this obesity–sepsis–PD-L1 relationship is inferred from obesity-associated immunology studies rather than demonstrated in septic patients. In murine sepsis, inhibition of PD-L1 restored monocyte function—marked by enhanced Major Histocompatibility Complex class II (MHC-II) expression—and reduced cytokine dysregulation (IL-6, TNF-α, IL-10), supporting PD-L1 as a driver of immune dysfunction [42]. Together, these findings suggest that MSCs mitigate sepsis by rebalancing immune responses, enhancing antimicrobial activity, and supporting tissue repair. This multifaceted activity distinguishes MSCs from conventional anti-inflammatory therapies and underscores their potential as innovative candidates for sepsis treatment in the pro-inflammatory phase.

It is important to note, that there is a conceptual duality in the role of PD-L1 during the stages of sepsis. In the early hyperinflammatory phase, PD-L1 upregulation—including on MSCs—may be beneficial by attenuating excessive immune activation and limiting tissue injury. In contrast, during the late immunosuppressive phase, sustained PD-1/PD-L1 signaling contributes to immune paralysis, monocyte dysfunction, and impaired pathogen clearance, and PD-L1 blockade can partially restore immune responsiveness [2,42]. However, a more complete understanding of MSCs across the entire disease spectrum, including both the hyperinflammatory and immunosuppressive phase, is required to tailor their therapeutic use in a clinical setting.

## 6. PD-L1 as a Biomarker for MSC Potency and Clinical Efficacy

The clinical translation of MSC therapies requires robust and reproducible methods to assess cell potency, predict therapeutic efficacy, and ensure product consistency. Among the various immunomodulatory molecules expressed by MSCs, PD-L1 has emerged as a promising biomarker candidate due to its central role in immune regulation and its inducibility under inflammatory conditions. Single-cell transcriptomic profiling has validated PD-L1 as a predictive marker for MSC immunosuppressive potency and clinical efficacy in autoimmune hepatitis [34]. Moreover, engineering MSCs to co-express PD-L1 and ICAM1 further enhances therapeutic outcomes in preclinical models [43]. 

### 6.1. Functional Biomarker of Immunosuppressive Potency

PD-L1 expression correlates strongly with the immunosuppressive capacity of MSCs. Guan et al. (2018) demonstrated that PD-L1 and indoleamine 2,3-dioxygenase 1 (IDO1) levels in IFN-γ-licensed MSCs were predictive of their ability to suppress T cell proliferation [1]. This highlights the need for a flow cytometry-based potency assay to measure intracellular PD-L1 and IDO1 as surrogate markers of MSC function. However, such PD-L1- and IDO1-based potency assays have not yet been validated across multiple donors or tissue sources, and it remains unclear how and if PD-L1 expression remains stable across passages and culture conditions. Such assays could offer a rapid, scalable, and mechanistically relevant approach to quality control in MSC manufacturing and important insight needed to maintain standardized manufacturing workflows.

Strauch, V et al. (2020) [18] further emphasized the importance of inflammatory licensing in enhancing PD-L1 expression and immunosuppressive function. Their study showed that cytokine-primed MSCs exhibited increased PD-L1 glycosylation and surface expression, which correlated with enhanced suppression of T cell activation [18]. These findings support the use of PD-L1 as a dynamic biomarker that reflects the immunological readiness of MSCs.

### 6.2. Predictive Value in Clinical Settings

Beyond manufacturing, PD-L1 expression may serve as a predictive biomarker for clinical efficacy. Li et al. (2021) [36] reported that patients with GvHD who received MSC therapy exhibited a rapid increase in circulating PD-L1^+^ sEVs. This increase correlated with reduced inflammatory markers and improved clinical outcomes, suggesting that PD-L1 levels may be used to monitor treatment response and guide dosing strategies [36]. 

In transplantation models, Mardomi et al. (2021) found that PD-L1-overexpressing MSCs were more effective in preventing graft rejection and promoting immune tolerance [37]. In future clinical trial designs, PD-L1 measurements could be incorporated both for donor selection—by prioritizing MSC products with defined PD-L1 profiles—and as an early on-treatment biomarker to monitor pharmacodynamic responses, adjust dosing strategies, or identify patients most likely to benefit from MSC therapy.

### 6.3. Standardization, Regulatory Consideration and (Lack of) Reference Distributions of PD-L1 in MSCs

Despite its promise, the use of PD-L1 as a biomarker faces several interrelated scientific, technical, and regulatory challenges. PD-L1 expression is highly context-dependent, influenced by donor variability, tissue source, culture conditions, and priming protocols. This variability complicates efforts to standardize measurement techniques, establish threshold levels for potency, and define biologically meaningful expression categories such as PD-L1^high^ and PD-L1^low^ subpopulations. 

From a regulatory perspective, agencies require biomarkers to be validated across multiple batches and clinical indications. Guan et al. (2018) emphasized the need for harmonized protocols and inter-laboratory reproducibility to support the inclusion of PD-L1 in MSC release criteria [1]. Moreover, integration of PD-L1 assays into good manufacturing practice (GMP) workflows must be streamlined to avoid delays and ensure compliance.

A major limitation is the absence of systematic human data defining baseline PD-L1 expression distributions in MSCs under steady-state conditions. The relative proportions of PD-L1^high^ versus PD-L1^low^ MSC subpopulations in healthy individuals have not been established in a standardized or comparable manner, restricting the ability to contextualize disease-associated changes. Consequently, reference ranges needed to interpret shifts in PD-L1 expression in pathological contexts, such as autoimmune disease, GvHD, sepsis, or post-transplant settings, remain lacking.

Preclinical research has strengthened the rationale for focusing on PD-L1, consistently demonstrating that elevated PD-L1 expression enhances MSC immunosuppressive function and therapeutic efficacy. Studies such as Bai et al. (2023) and Lou et al. (2025) show that PD-L1^high^ MSCs outperform PD-L1^low^ counterparts, supporting PD-L1 as a functional biomarker of MSC potency [34,43]. Additionally, a substantial body of preclinical work has also explored mechanisms to induce PD-L1 expression in MSCs and MSC-derived extracellular vesicles across both in vitro and in vivo systems [36]. However, clinical translation of these findings remains limited, as existing human studies primarily assess inducible PD-L1 expression following inflammatory licensing (e.g., IFN-γ, TNF-α), rather than resolving stable or disease-specific subpopulation structures at single-cell resolution. Notably, no clinical studies to date have prospectively stratified or engineered MSC products based on PD-L1 subpopulation composition, nor correlated these distributions with therapeutic outcomes in patients.

Addressing these combined challenges will require the development of standardized, quantitative, and single-cell-resolved frameworks to define PD-L1 expression in human MSCs. Establishing robust assays, reproducible reference distributions, and clearly defined disease-associated deviations will be essential for transitioning PD-L1 from a context-dependent marker to a validated and regulatory-acceptable potency attribute in clinical MSC manufacturing.

### 6.4. PD-L1 in the Context of Established MSC Potency Markers

MSC immunomodulatory potency is mediated by a complex network of surface molecules, soluble factors, and metabolic enzymes, including indoleamine 2,3-dioxygenase (IDO1), prostaglandin E2 (PGE2), transforming growth factor-β (TGF-β), nitric oxide, and additional checkpoint ligands. Within this framework, PD-L1 represents a mechanistically distinct class of potency marker, functioning as a cell-surface immune checkpoint with direct relevance to T-cell exhaustion, anergy, and regulatory T-cell induction. Compared with soluble mediators such as PGE2 or TGF-β, PD-L1 offers practical and analytical advantages, including direct quantification by flow cytometry and single-cell resolution, enabling the assessment of cellular heterogeneity within MSC products. In comparison, markers such as IDO1 and PGE2 primarily reflect downstream metabolic activity and are often influenced by culture conditions and the duration of inflammatory licensing.

Given the complexity of MSC immunomodulation, PD-L1 is unlikely to function as a standalone potency biomarker. Instead, its greatest translational value lies in its integration into multi-parameter potency panels that incorporate surface checkpoint expressions, metabolic activity (e.g., IDO1, PGE2), immunoregulatory cytokines (e.g., TGF-β), and functional suppression assays. Such composite approaches provide a more comprehensive assessment of MSC activity and improve the predictive accuracy of therapeutic outcomes [4,44]. The application of machine learning models and bioinformatics tools to these multi-dimensional biomarker datasets may further enhance the utility of PD-L1 in MSC characterization. By identifying complex patterns and correlations not apparent through single-marker analyses, these approaches may enable more refined potency assessment and support the development of personalized MSC therapies.

## 7. Mechanistic Insights and Molecular Interactions

The immunomodulatory effects of MSCs are mediated through a complex network of molecular interactions, with PD-L1 playing a central role. Understanding the mechanistic basis of PD-L1 function in MSCs provides insight into how these cells orchestrate immune suppression and tolerance. This section explores the crosstalk between PD-L1 and other immune checkpoints, its influence on cytokine signaling, and the emerging role of exosomal PD-L1 in cell-free immunomodulation. 

### 7.1. Crosstalk with Other Immune Checkpoints

PD-L1 does not act in isolation. It is part of a broader immune checkpoint landscape that includes molecules such as Cytotoxic T-Lymphocyte-Associated protein 4 (CTLA-4), TIM-3, Lymphocyte Activation Gene 3 (LAG-3), and T cell Immunoreceptor with Ig and ITIM domains (TIGIT). Zhou et al. (2018) [3] demonstrated that AD-MSCs suppress T cell subsets via both PD-L1/PD-1 and Galectin-9/TIM-3 pathways. This dual engagement enhances the immunosuppressive effect and promotes T cell exhaustion, particularly in chronic inflammatory environments [3]. 

In small-cell-lung-cancer models, Kursunel et al. (2022) showed that cancer stem-like MSCs upregulate PD-L1, PD-L2, and other checkpoint ligands in response to IFN-γ secreted by T cells [45]. This feedback loop contributes to immune evasion and highlights the importance of checkpoint synergy in MSC-mediated regulation. 

The expression of multiple checkpoint ligands by MSCs suggests that combinatorial targeting may be necessary to fully reverse immune suppression in certain contexts. It also opens the possibility of engineering MSCs to selectively express or silence specific checkpoints depending on therapeutic goals.

### 7.2. Cytokine Modulation and Feedback Loops

PD-L1 expression in MSCs is tightly linked to cytokine signaling. Inflammatory cytokines such as IFN-γ, TNF-α, and IL-1β upregulate PD-L1 through STAT1 and NF-κB pathways [15,18]. This upregulation enhances MSC immunosuppressive function and creates a feedback loop in which immune activation leads to immune regulation. 

Behm et al. (2020) [4] showed that periodontal ligament-derived MSCs exposed to cytokines increased PD-L1, IDO1, and PGE2 expression, resulting in suppression of CD4^+^ T cell proliferation and induction of apoptosis. These effects were cytokine-specific, with IFN-γ and IL-1β having the strongest impact [4]. 

Moreover, MSCs themselves secrete cytokines that influence PD-L1 expression on neighboring immune cells. Eljaafari et al. (2021) reported that AD-MSCs from obese individuals secreted IFN-γ and IL-6, which induced PD-L1 expression in white adipose tissue and contributed to T cell dysfunction [6]. This paracrine signaling underscores the systemic impact of MSCs on immune regulation.

### 7.3. Exosomal PD-L1 and Cell-Free Immunomodulation

Exosomes are sEVs that carry proteins, lipids, and nucleic acids. MSC-derived exosomes have emerged as potent immunomodulatory agents, capable of recapitulating many of the effects of their parent cells. PD-L1 is among the key molecules found on MSC-derived exosomes, and its presence has functional consequences [13,23]. 

Li et al. (2021) [36] demonstrated that PD-L1-enriched exosomes from WJ-MSCs suppressed T cell activation and promoted regulatory phenotypes. These exosomes were rapidly detectable in circulation following MSC infusion and correlated with clinical improvement in GvHD [36]. 

The use of exosomal PD-L1 offers several advantages: it enables cell-free therapy, reduces the risk of cell engraftment complications, and allows for targeted delivery. However, it also raises concerns about off-target effects and long-term immune suppression, particularly in cancer or chronic infection settings. 

Recent studies have explored engineering MSC-derived exosomes to enhance PD-L1 expression or combine it with other immunomodulatory molecules [36]. These approaches may improve therapeutic efficacy while allowing for precise control over immune modulation.

## 8. Challenges and Future Directions

While the therapeutic potential of PD-L1-expressing MSCs is increasingly recognized, several challenges must be addressed to ensure safe, effective, and standardized clinical applications. These challenges span biological variability, safety concerns, regulatory hurdles, and the need for personalized approaches. 

Several of the strategies discussed in this section—including genetic or epigenetic engineering of MSCs, exosome-based cell-free platforms, and combinatorial checkpoint modulation—are supported predominantly by mechanistic and preclinical evidence. While these approaches compellingly demonstrate that enhanced PD-L1 expression is biologically sufficient to augment MSC-mediated immunoregulation, they cannot be interpreted as absolute evidence that endogenous PD-L1^low^ MSC populations may presently be identified, corrected, or therapeutically rebalanced in human disease.

Accordingly, these strategies are best viewed as proof-of-concept explorations that reinforce the central role of the PD-1/PD-L1 axis in MSC biology, rather than as clinically validated interventions. Clarifying how naturally occurring PD-L1 expression heterogeneity arises in vivo, and whether it can be meaningfully modified in patients, remains an unmet prerequisite for translation.

### 8.1. Safety Concerns and Tumor Immunity

One of the most pressing concerns regarding PD-L1-enhanced MSC therapies is the potential for promoting tumor immune evasion. PD-L1 is a well-established mechanism by which cancer cells suppress cytotoxic T cell responses and escape immune surveillance [5]. While MSCs are not inherently tumorigenic, their immunosuppressive properties, particularly when amplified by PD-L1 overexpression, could theoretically support tumor growth or metastasis in susceptible individuals. 

This risk is especially relevant in patients with latent malignancies or those undergoing MSC therapy for inflammatory conditions associated with cancer risk. Careful screening, dose control, and monitoring of PD-L1 levels are essential to mitigate this concern. Moreover, long-term studies are needed to evaluate the oncogenic potential of PD-L1-enhanced MSCs in vivo.

### 8.2. Safety Concerns and Viral Immune Evasion

In addition to oncogenic risks, PD-L1-enhanced MSC therapies may also impair antiviral immunity. The PD-1/PD-L1 axis, though central to immune tolerance, is also a well-established mechanism of viral immune evasion, particularly in chronic infections such as human immunodeficiency virus (HIV), hepatitis B virus (HBV), hepatitis C virus (HCV), and latent herpesviruses such as cytomegalovirus (CMV), Epstein–Barr virus (EBV), and herpes simplex virus (HSV). High PD-L1 expression can contribute to T cell exhaustion, reducing the effectiveness of cytotoxic responses and allowing latent viruses to reactivate. This phenomenon has been observed in both cancer and infectious disease models, where PD-L1 signaling suppresses antiviral cytokine production (e.g., IFN-γ, IL-2) and impairs viral clearance. 

In the context of MSC therapy, especially when cells are IFN-γ-primed to enhance PD-L1 expression, this immunosuppressive effect may inadvertently worsen or reactivate underlying viral infections [46]. Phases of immunosuppression and T-cell exhaustion may also allow for opportunistic infections to take hold over a patients weakened immune system. As Keane et al. (2017) noted, seventy percent of sepsis deaths now occur in this phase, which is characterized by opportunistic pathogen superinfections, latent viral reactivation, and evidence for profound immunosuppression [40]. 

It is important to emphasize that these outcomes remain hypothetical but potentially serious, and further investigation is needed to determine whether MSCs possess intrinsic regulatory mechanisms—such as a “safety stop”—that prevent excessive immune suppression. To reduce the risk of viral reactivation and opportunistic infections, careful preclinical evaluation is warranted, including assessment of latent infection status, stratification of susceptibility, and longitudinal monitoring of virologic parameters during and after MSC administration.

### 8.3. Donor Variability and MSC Source

MSC characteristics, including PD-L1 expression, exhibit substantial variation influenced by multiple intrinsic and extrinsic factors such as: donor characteristics (age, sex, health status), tissue origin (e.g., bone marrow, adipose tissue, umbilical cord) and culture conditions (oxygen tension, passage number, cytokine priming) [47,48,49,50].

Selle et al. (2022) found that the PD-L1 expression on humane BM-MSCs decreased with increasing donor age, with a more pronounced reduction observed among female donors [47]. Similarly, Bai et al. (2023) studied WJ-MSCs isolated from 58 donors and identified marked inter-donor variability in the expression levels of PD-L1, emphasizing the inherent heterogeneity among MSC populations [34]. This biological variability complicates standardization of MSC products and may affect therapeutic outcomes [34,44]. 

To address this, researchers are exploring the use of clonal MSC lines, genetically engineered MSCs, and defined priming protocols to reduce heterogeneity. Dunn et al. (2022) demonstrated that IFN-γ-primed clonal MSC sheets exhibit consistent PD-L1 expression and enhanced immunosuppressive function, suggesting a path forward for reproducible cell products [44]. 

Notably, the majority of studies investigating MSC biology and therapeutic potential have relied on single-source MSCs, primarily aiming to characterize biological differences across distinct tissue origins. However, direct comparative analyses between MSCs derived from different sources remain limited.

### 8.4. Regulatory and Manufacturing Challenges

The integration of PD-L1 as a biomarker in MSC manufacturing requires validation, regulatory approval, and harmonization across laboratories. Guan et al. (2018) emphasized the need for standardized assays, reference materials, and inter-laboratory reproducibility to support PD-L1-based potency testing [1]. 

Regulatory agencies such as the FDA and EMA require biomarkers to be linked to clinical outcomes and validated in multiple settings. This necessitates large-scale clinical trials, robust data collection, and collaboration between academic, industry, and regulatory stakeholders. 

Moreover, the manufacturing of MSCs under good manufacturing practice (GMP) conditions must accommodate PD-L1 testing without compromising workflow efficiency. Incorporating automation, digital tracking systems, and real-time analytics into quality management systems may help streamline this process. These tools can support compliance with GMP standards while maintaining assay accuracy, consistency, and reliability.

### 8.5. Personalized MSC Therapies

Given the complexity of immune responses and the variability of MSCs, personalized approaches may enhance therapeutic efficacy. This includes tailoring MSC products based on patient immune profiles, disease stage, and biomarker expression. For example, patients with high levels of PD-1^+^ T cells may benefit more from PD-L1-enriched MSCs, while those with exhausted immune systems may require alternative strategies. 

Advanced analytics, including machine learning and multi-omics profiling, can support patient stratification and product customization. These tools can identify predictive biomarkers, optimize dosing, and monitor treatment responses in real time.

### 8.6. Future Research Directions

Several promising avenues are emerging to enhance the safety, efficacy, and translational potential of PD-L1-enhanced MSC therapies. These directions span molecular engineering, delivery innovations, and mechanistic studies aimed at optimizing therapeutic outcomes.

#### 8.6.1. Engineering MSCs for Enhanced Immunomodulation

Genetic modification of MSCs to overexpress PD-L1 or co-express it with other immunoregulatory molecules (e.g., IL-10, TGF-β) may improve their therapeutic efficacy. Notably, MSCs engineered to co-express PD-L1 and ICAM1 (PI-MSCs) have demonstrated superior homing capabilities and immunoregulatory function in preclinical models [51]. Future work should explore combinatorial engineering strategies to fine-tune immune modulation while minimizing off-target effects.

#### 8.6.2. PD-L1 and Hypoimmunogenic Cell Engineering Strategies

The increasing focus on PD-L1 in MSC immunobiology aligns with broader efforts in regenerative medicine to develop hypoimmunogenic or immune-evasive cell products, particularly for allogeneic applications. In stem cell–derived therapies, strategies such as immune checkpoint augmentation, attenuation of antigen presentation pathways, and modulation of NK-cell recognition have been explored to prolong graft persistence and reduce host immune rejection [52].

Within this broader landscape, PD-L1 overexpression in MSCs represents a targeted and biologically grounded approach that exploits an endogenous immunoregulatory pathway rather than wholesale immune invisibility. This may confer advantages in safety and controllability, particularly when compared with more extensive genetic modifications aimed at eliminating immune recognition altogether.

These observations position PD-L1 not merely as an MSC-specific feature, but as part of a generalizable framework for designing next-generation cell therapies with enhanced immune compatibility.

#### 8.6.3. Exosome-Based, Cell-Free Therapeutic Platforms

MSC-derived exosomes carrying PD-L1 represent a promising cell-free alternative that may reduce the risks associated with live cell therapies. These vesicles retain immunomodulatory properties and offer advantages in terms of stability, targeting, and scalability [36]. When looking at it from a production standpoint, the use of exosome-based therapy offers some advantages as well in form of easier and longer storage and a more standardized and reproducible product. Further research is needed to optimize exosome isolation, loading, and delivery, and to evaluate their pharmacokinetics and biodistribution in vivo.

#### 8.6.4. Synergistic Checkpoint Modulation

The interplay between PD-L1 and other immune checkpoints—such as TIM-3, LAG-3, and CTLA-4—remains underexplored in the context of MSC therapy. Combinatorial checkpoint modulation may unlock synergistic effects that enhance immune tolerance or resolve chronic inflammation [3]. Future studies should investigate dual or multiplex checkpoint strategies, potentially delivered via engineered MSCs or exosomes [13].

#### 8.6.5. Longitudinal and Mechanistic Studies

The majority of studies in the field of MSC research have focused on the preclinical characterization and data accumulation of MSCs derived from single-tissue sources. Comparative investigations assessing MSCs from different origins and delineating their biological and functional differences remain relatively scarce. Further studies directly comparing MSCs from diverse sources are therefore essential to elucidate source-dependent variability and its implications for therapeutic efficacy and standardization. To ensure long-term safety and efficacy, it is critical to track PD-L1 expression dynamics and immune responses over time. Longitudinal studies in relevant disease models and clinical cohorts will help clarify the durability of immunosuppression, potential for immune escape, and risk of tumorigenesis. These studies should integrate immune phenotyping, cytokine profiling, and tissue-specific analyses to build a comprehensive understanding of MSC-host interactions. To complement these perspectives, an overview of the immunomodulatory effects of PD-L1-expressing MSCs across immune cell populations is provided in Table 1.

## 9. Conclusions

PD-L1 expression in MSCs represents a pivotal mechanism by which these cells exert immunomodulatory effects on both innate and adaptive immune systems. Several mechanisms are now firmly established, including IFN-γ–STAT1-mediated induction of PD-L1, its central role in suppressing effector T-cell activation and promoting regulatory subsets, and the translational potential of circulating PD-L1^+^ sEVs in GvHD as an early pharmacodynamic marker. These properties have been harnessed in a range of therapeutic applications, including autoimmune diseases, GvHD, transplantation, and sepsis. However, important gaps remain: the contribution of PD-L1 to immune paralysis in the late immunosuppressive phase of sepsis requires further clarification; the distribution of PD-L1^high^ and PD-L1^low^ MSC subpopulations in healthy individuals and across disease contexts remains poorly defined; and direct evidence for MSC–NK PD-1/PD-L1 signaling is still limited. Addressing these uncertainties will be essential to refine patient stratification and therapeutic timing.

The inducibility of PD-L1 under inflammatory conditions, its integration with other immune checkpoints, and its presence on MSC-derived exosomes underscore its versatility and importance. Moreover, PD-L1 has emerged as a promising biomarker for MSC potency and clinical efficacy, offering a mechanistically relevant and scalable tool for product characterization and patient stratification.

It is important to distinguish between the strong preclinical evidence supporting PD-L1-dependent MSC immunomodulation and the limited clinical data currently available. While animal models and in vitro human systems consistently demonstrate that PD-L1 expression correlates with enhanced suppression of pathological immune responses, clinical studies in GvHD, autoimmune disease, transplantation, and sepsis have largely inferred PD-L1 involvement indirectly, without systematic stratification or outcome correlation. 

Bridging this gap of evidence will require prospective human studies that integrate PD-L1 expression profiling with clinical endpoints, ideally within standardized manufacturing and potency assessment frameworks.

Despite its promise, PD-L1-enhanced MSC therapy faces challenges related to safety, standardization, and regulatory approval. The potential for tumor immune evasion, donor variability, and context-dependent effects necessitates careful evaluation and the development of personalized therapeutic strategies. Future research should focus on engineering MSCs for optimized checkpoint expression, developing multi-parameter GMP-compatible potency assays, and conducting longitudinal studies to assess durability and safety. In addition, clinical priorities should include incorporating PD-L1 measurements—both cellular and sEV-associated—into prospective trials, performing comparative studies of PD-L1^high^ versus PD-L1^low^ MSC products across tissue sources, and further dissecting the role of PD-L1 in late-phase sepsis and chronic infection. In summary, PD-L1 is not only a key effector molecule in MSC-mediated immune regulation but also a strategic target for enhancing the therapeutic utility of MSCs. Its integration into clinical protocols and manufacturing workflows will be essential for advancing MSC-based therapies and improving outcomes in a broad spectrum of immune-mediated diseases.

## Figures and Tables

**Table 1:** Effects of PD-L1 Expression in MSCs on Immune System Components.

Immune Component	Effect of PD-L1 Expression in MSCs	Mechanism	Outcome	Reference
CD4^+^ T cells	Suppression of proliferation and cytokine production	PD-L1/PD-1 interaction inhibits NF-κB signaling	Reduced inflammation, promotion of tolerance	[8,14]
CD8^+^ T cells	Inhibition of cytotoxic activity	PD-L1 binding to PD-1 reduces IFN-γ and granzyme B secretion	Prevention of tissue damage, immune regulation	[8,53]
Regulatory T cells (Tregs)	Expansion and functional enhancement	PD-L1 promotes FoxP3^+^ Treg differentiation	Maintenance of immune homeostasis	[1]
Th17 cells	Suppression of differentiation and IL-17 production	PD-L1 engagement reduces STAT3 activation	Attenuation of autoimmune responses	[54,55]
T cell exhaustion	Induction or reversal depending on context	PD-L1 in chronic inflammation and sepsis promotes T cell exhaustion	Impaired immune responsiveness	[2,3]
B cells	Inhibition of activation and antibody production	PD-L1/PD-1 binding directly induces the differentiation of B cells to IL-10 producing Breg cells	Reduced autoantibody levels, increased Bregs cell numbers	[31,32]
Regulatory B cells (Bregs)	Promotion of IL-10-secreting Bregs	PD-L1 and cytokine milieu (e.g., IL-10, TGF-β)	Enhanced immune tolerance	[32]
Dendritic cells (DCs)	Impaired maturation and antigen presentation	PD-L1 suppresses co-stimulatory molecules (CD80/CD86)	Reduced T cell priming and activation	[8,20,21]
Monocytes	Selective apoptosis of pro-inflammatory subsets	PD-L1-enriched MSC-derived exosomes target CD14^+^CD16^+^ monocytes	Shift toward anti-inflammatory monocyte populations	[24]
Macrophages	Polarization toward M2 (anti-inflammatory) phenotype	PD-L1/PD-1 interaction induces CD206 and IL-10 secretion by promoting metabolic reprogramming and mitochondrial function	Enhanced tissue repair and resolution of inflammation	[23,56]
Natural Killer (NK) cells	Suppression of cytotoxicity and cytokine release	PD-L1/PD-1 interaction reduces activation markers and effector molecules	Prevention of excessive immune responses	[6,29,30]
Immune checkpoints	Synergistic regulation with TIM-3, CTLA-4, LAG-3	Co-expression and engagement of multiple inhibitory receptors	Enhanced immunosuppressive effect and promotes tolerance	[3]
Exosomal PD-L1	Cell-free immunosuppression	PD-L1 carried on MSC-derived exosomes suppresses T cell activation and promotes regulatory phenotypes remotely	Potential for targeted, non-cell-based therapies	[36]

## Data Availability

No new data were created or analyzed in this study. Data sharing is not applicable to this article.

## Abbreviations

The following abbreviations are used in this manuscript:

Breg	Regulatory B cell
CMV	Cytomegalovirus
CTLA-4	CTLA-4
DC	Dendritic cell
EAE	Experimental autoimmune encephalomyelitis
EBV	Epstein–Barr virus
GvHD	Graft-versus-host disease
HBV	Hepatitis B virus
HCV	Hepatitis C virus
HIV	Human immunodeficiency virus
HSV	Herpes simplex virus
IDO	Indoleamine 2,3-dioxygenase
IFN-γ	Interferon-gamma
IL-1β	Interleukin-1 beta
IL-6 –	Interleukin-6
IRF1	Interferon regulatory factor 1
JAK	Janus Kinase
LAG-3	Lymphocyte activation gene 3
MHC-II	Major histocompatibility complex class II
MSC	Mesenchymal stem/stromal cell
NF-κB	Nuclear factor kappa-light-chain-enhancer of activated B cells
NK cell	Natural killer cell
PD-1	Programmed death-1
PD-L1	Programmed death-ligand 1
PGE2	Prostaglandin E2
PRRX1	Paired related homeobox 1
sEV	Small extracellular vesicle
sPD-L1	Soluble programmed death-ligand 1
STAT1/3	Signal transducer and activator of transcription 1/3
TGF-β	Transforming growth factor-beta
TIGIT	T-cell immunoreceptor with Ig and ITIM domains
TIM-3	T-cell immunoglobulin and mucin-domain containing-3
TNF-α	Tumor necrosis factor-alpha
Treg	Regulatory T cell
WJ-MSC	Wharton’s Jelly-derived mesenchymal stem/stromal cell
